# Neuroethology of Olfactory-Guided Behavior and Its Potential Application in the Control of Harmful Insects

**DOI:** 10.3389/fphys.2016.00271

**Published:** 2016-06-30

**Authors:** Carolina E. Reisenman, Hong Lei, Pablo G. Guerenstein

**Affiliations:** ^1^Department of Molecular and Cell Biology and Essig Museum of Entomology, University of California, BerkeleyBerkeley, CA, USA; ^2^Department of Neuroscience, University of ArizonaTucson, AZ, USA; ^3^Lab. de Estudio de la Biología de Insectos, CICyTTP-CONICETDiamante, Argentina; ^4^Facultad de Ingeniería, Universidad Nacional de Entre RíosOro Verde, Argentina

**Keywords:** crop pest, disease vector, integrated pest management, odor attractant, disruption of behavior, odor repelllent, insect neuroethology

## Abstract

Harmful insects include pests of crops and storage goods, and vectors of human and animal diseases. Throughout their history, humans have been fighting them using diverse methods. The fairly recent development of synthetic chemical insecticides promised efficient crop and health protection at a relatively low cost. However, the negative effects of those insecticides on human health and the environment, as well as the development of insect resistance, have been fueling the search for alternative control tools. New and promising alternative methods to fight harmful insects include the manipulation of their behavior using synthetic versions of “semiochemicals”, which are natural volatile and non-volatile substances involved in the intra- and/or inter-specific communication between organisms. Synthetic semiochemicals can be used as trap baits to monitor the presence of insects, so that insecticide spraying can be planned rationally (i.e., only when and where insects are actually present). Other methods that use semiochemicals include insect annihilation by mass trapping, attract-and- kill techniques, behavioral disruption, and the use of repellents. In the last decades many investigations focused on the neural bases of insect's responses to semiochemicals. Those studies help understand how the olfactory system detects and processes information about odors, which could lead to the design of efficient control tools, including odor baits, repellents or ways to confound insects. Here we review our current knowledge about the neural mechanisms controlling olfactory responses to semiochemicals in harmful insects. We also discuss how this neuroethology approach can be used to design or improve pest/vector management strategies.

## Introduction

Humans benefit from insects, mainly as pollinators of crops, but an important number of other insects are pests of crops or damage storage goods, are vectors of serious human and animal diseases, or are simply a nuisance. For centuries, humans have been fighting harmful insects, and the use of synthetic or genetically modified plant-produced chemical insecticides has made this fight much more efficient. However, the use and overuse of those chemicals has led to a number of undesirable consequences, such as contamination of our environment, food and water, and insecticide resistance. In addition, the rising of the organic agriculture movement demands insecticide-free food (van der Goes van Naters and Carlson, 2006).

Chemicals other than insecticides can be used to fight insects through the manipulation of specific olfactory behaviors, profiting from the existence of natural compounds used for communication between organisms, the semiochemicals (Pickett et al., 1997). Pheromones are perhaps the most well-known class of semiochemicals. Pheromones mediate interactions between organisms of the same species, and include, sex, aggregation, and alarm substances, while allelochemicals are semiochemicals that mediate inter-specific interactions (see Dusenbery, 1992; Wyatt, 2003 for further details).

The potential use of semiochemicals to monitor, disrupt, lure, repel, confuse, or mass-trap insect pests was rapidly acknowledged and has fueled much research (Wyatt, 2003; Witzgall et al., 2010) with the promise of clean, safe, and highly specific pest and vector control tools. For instance, mating disruption, in which large amounts of a synthetic sex pheromone are released in a crop, has been used to eradicate insect pests that became resistant to pesticides (Wyatt, 2003; Witzgall et al., 2010). Semiochemicals can also be used for trapping insects in integrated pest and vector control management strategies. Thus, when trapping devices include insecticides, insects attracted to a semiochemical also pick up lethal substances or pathogens (a strategy known as “lure and kill”; Pickett et al., 1997; Wyatt, 2003).

In the last decades, many studies focused on the neural mechanisms underlying behavioral responses to semiochemicals. These investigations aid the design of odor-based strategies for insect control, as they help understanding how the olfactory system processes information about odors and also allow generating predictions about the insect's olfactory behavior (e.g., Hildebrand, 1996; Guerenstein and Hildebrand, 2008). Unfortunately, research in the fields of neuroethology and insect control has been often segregated, which may hamper the development of novel and efficient control tools and strategies. In light of this, here we review our current knowledge about the neural mechanisms controlling olfactory responses to semiochemicals in harmful insects, and also discuss how this neuroethology approach can be used to manipulate insect behavior and therefore improve pest/vector management strategies. We start by briefly summarizing the structure and function of the insect olfactory system.

## The insect olfactory system

Olfactory receptor cells (ORCs) are the first neural elements in the olfactory pathway and are housed in variable numbers in hair-like, multi-porous structures known as olfactory sensilla. Olfactory sensilla are located mainly on the antennae and in some insects also in the mouthparts. After entering the sensillum through its wall pores, odors diffuse in the aqueous sensillum lymph (sometimes transported by odorant binding proteins, Vogt and Riddiford, 1981; Tsuchihara et al., 2005; Leal, 2013) and reach the dendrites of the ORCs. There, odors interact with different classes of chemoreceptor proteins: odorant receptors (ORs), ionotropic receptors (IRs), or gustatory receptors (GRs; Vosshall et al., 1999; Larsson et al., 2004; Vosshall and Stocker, 2007; Vosshall and Hansson, 2011; Suh et al., 2014). Many ORCs respond to only one or a few related odor compounds, particularly when tested at behaviorally relevant and naturally-occurring concentrations, but others are more broadly tuned (e.g., de Bruyne et al., 1999; Hansson et al., 1999; Stranden et al., 2003; Yao et al., 2005; Hallem and Carlson, 2006; Martelli et al., 2013). In all cases their response spectra depends on the odor tuning of the chemoreceptor protein/s expressed (e.g., Hallem and Carlson, 2006; Andersson et al., 2015). Each type of ORC usually expresses only one type of OR, IR, or GR (e.g., Vosshall et al., 1999; Galizia and Sachse, 2010). However, in some ORCs more than one OR, IR, or GR types, and even different chemoreceptor protein types (most commonly ORs and IRs), are co-expressed, and in those cases odors interact with more than one chemoreceptor protein type (e.g., Fishilevich and Vosshall, 2005; Abuin et al., 2011; Rytz et al., 2013; Hussain et al., 2016; see below).

Odorant receptors are usually expressed in ORCs within single-walled (basiconic or trichoid) sensilla. They are part of a heteromeric complex consisting of an OR-subunit which binds the odor ligand (thus conferring odor specificity) and the highly conserved OR co-receptor (ORCO). Experimental evidence suggests alternative mechanisms of odor activation, one in which OR-ORCO forms a non-selective ligand-activated cation channel, and the other in which ORCO itself functions as a cation channel (Sato et al., 2008; Wicher et al., 2008). Although ORCO orthologs exist in many insect species, to date there is no agreement on how ORCO functions during olfactory transduction *in vivo* (Stengl and Funk, 2013).

ORCs that respond to compounds such as ammonia, short chain carboxylic acids and amines are housed in double-walled (grooved peg and coeloconic) sensilla (Pappenberger et al., 1996; Diehl et al., 2003; Benton et al., 2009; Hussain et al., 2016) and do not express ORs but instead IRs. The IRs form ionic channels activated by ligands (Benton et al., 2009) and are expressed with one or two broadly expressed co-receptors different from ORCO (Abuin et al., 2011; Ai et al., 2013; Rytz et al., 2013). In addition, the very volatile molecule CO_2_, which is of primordial importance for the olfactory orientation of blood-sucking insects and some herbivores (Guerenstein and Hildebrand, 2008), is detected by two to three members of the GR family co-expressed in a single ORC type (Suh et al., 2004; Jones et al., 2007; Kwon et al., 2007; Lu et al., 2007; Kent et al., 2008; Wang et al., 2013).

The axons of the ORCs project to the first processing center of olfactory information in the insect brain, the antennal lobe (AL; e.g., Anton and Homberg, 1999). The AL, analogous to the vertebrate olfactory bulb, is composed of distinct spheroid structures called glomeruli (Anton and Homberg, 1999; Fishilevich and Vosshall, 2005). Usually, the terminals of ORCs expressing the same chemoreceptor protein converge onto a single glomerulus (Vosshall et al., 2000; Guerenstein et al., 2004a; Rytz et al., 2013; Suh et al., 2014; Hussain et al., 2016). Each glomerulus also houses neurites of local interneurons (LNs) and of projection neurons (PNs). LNs are restricted to the AL and have dendritic arborizations in several glomeruli; PNs usually arborize in one glomerulus and have an axon that projects to higher brain areas in the protocerebrum such as the lateral horn, the inferior lateral PC, and the calyces of the ipsilateral mushroom body (Homberg et al., 1988, 1989; Jefferis et al., 2007; Galizia and Rössler, 2010; Tanaka et al., 2012; Roussel et al., 2014). Neurons in these higher-order brain centers show diverse responses and integrate information about different odor compounds (e.g., Jefferis et al., 2007; Turner et al., 2008; Gupta and Stopfer, 2012; Lei et al., 2013); neurons receiving input from the mushroom body calyces are involved in mediating learning and memory processes (e.g., Davis, 2004; Fahrbach, 2006; Liu et al., 2012). Further downstream, circuits in the lateral accessory lobe and the ventral protocerebrum have been linked, particularly in moths, to important aspects of olfactory behaviors (e.g., Olberg, 1983; Iwano et al., 2010).

In the next sections we review current knowledge about the neural and behavioral mechanisms underlying responses to diverse classes of pheromones, host odors, and plant volatiles, mechanisms of olfactory repellence, disruption of olfactory behavior, and the effects of experience and learning in olfactory-driven behaviors.

## Olfactory attraction for monitoring and trapping

### Use of sex pheromones

Pheromones are usually mixtures of several compounds. Thus, the development of synthetic pheromone-blend attractants as trap lures involves knowledge of the compound identities, their concentrations, and their relative proportions. In several sympatric moth species, females release sex pheromones of overlapping chemical composition but with species-specific compound ratios, suggesting that males use this information to find conspecific females. For instance, different strains of the European corn borer (*Ostrinia nubilalis*) are attracted to precise pheromone blend ratios (Klun et al., 1973). Similarly, different species of *Yponomeuta* moths, which feed on the same host and share the same three pheromone constituents, are reproductively isolated due to differential attraction to species-specific blend ratios (Löfstedt et al., 1991). Similar findings were also reported on aphids (Dewhirst et al., 2010) and plant bugs (Byers et al., 2013). While the importance of ratios is crucial for the design of trap lures, the neural mechanisms underlying this phenomenon just began to be understood (e.g., Martin et al., 2013).

Sex pheromones can be used for monitoring and trapping many insect species. While we review and discuss what is known across different insect species, much is known about the neurobiological bases of mate seeking and finding in the moth *Manduca sexta*. Knowledge gained through studies in this insect could be applied to other insect-pest species, particularly other moths, as it is likely that similar neural mechanisms underlie mate odor-guided seeking behavior.

In moths and cockroaches, information about the female sex pheromone is processed by a small number of male-specific AL glomeruli forming a distinct structure, the macroglomerular complex (MGC; e.g., Boeckh and Boeckh, 1979; Hildebrand et al., 1980). Although the MGC sub-system of moths is distinctive and particularly large, the synaptic organization and structure of its constituent glomeruli is akin to that of the rest of the AL glomeruli. In some moth species, each MGC glomerulus processes a cognate pheromone component (e.g., *Heliothis virescens*; Berg et al., 1998), but in other species multiple components are encoded in the same MGC subcompartment (e.g., *Spodoptera littoralis*; Anton and Hansson, 1995). In other cases, pheromones and plant odorants are processed by the same MGC neurons (e.g., *Agrotis ipsilon*; Rouyar et al., 2015). Given this complexity, the use of simpler model systems (e.g., see next) can be experimentally advantageous and help the discovery of common, basic principles underlying the processing of complex odor blends.

The MGC of *M. sexta* has two main glomeruli, the Cumulus and the Toroid, each processing information about one of the two major female sex pheromone blend components (Hansson et al., 1991, 1992; Heinbockel et al., 1999). Because only these two components (out of eight total) are required to elicit odor-induced orientation behaviors in males (Tumlinson et al., 1989), this provides a simple binary system to investigate the neural mechanisms mediating pheromone processing, including blend ratio processing. When males are stimulated with the pheromone blend, two distinct populations of ORCs are specifically activated by those two essential components, one evoking excitatory responses in Cumulus projection neurons (cPNs) and the other in Toroid projection neurons (tPNs; Kaissling et al., 1989; Hansson et al., 1992; Hildebrand, 1996; Heinbockel et al., 1999; Lei et al., 2002). Additionally, recent findings suggest that cPNs and tPNs correlate their synaptic output to signal the presence of the pheromone blend (Lei et al., 2013; Martin et al., 2013). In principle, the odor-evoked spiking activity of cPNs and tPNs could serve to report the chemical identity and concentration of each blend component. However, since their outputs converge in the same regions in the protocerebrum (the delta region of the lateral horn and the mushroom body calyces), the relative timing of input spikes from cPNs and tPNs in postsynaptic neurons may have a physiological effect, that is, coincident spikes would evoke a stronger response in postsynaptic neurons than sequential spikes, allowing the representation of an odor mixture as a single odor object (see also Section Effects of Background Odor).

Indeed, using simultaneous dual-electrode intracellular recordings, Lei et al. (2002) showed inter- and intra-glomerular spike synchrony among PNs in response to pheromone blend stimulation. Odor-induced interglomerular synchrony in the AL was also reported in cockroaches using voltage-sensitive-dye imaging methods, suggesting that the synchrony code operates at a broad spatial scale (Watanabe, 2012). Moreover, experiments that simultaneously recorded neuronal activity across the glomerular array in *M. sexta* showed that neurons with the most similar odor response profiles produced the highest degree of coincident spikes (Lei et al., 2004). These results support the notion that PNs may use a correlative neural code. In addition, local field potential oscillations in the mushroom bodies, which are thought to reflect evolving ensemble synchrony of PNs across the entire array of AL glomeruli, were reported in many insect species, including locusts, fruit flies, and moths (MacLeod and Laurent, 1996; Ito et al., 2009; Tanaka et al., 2009). Further, it has been shown that spike coincidence in *M. sexta* AL neurons is modulated by the pheromone blend ratio. Behaviorally, the moths respond best to the mixture of the two essential pheromone components at the naturally occurring 1:2 ratio, and deviations from this ratio deteriorate blend attractiveness (Martin et al., 2013). By stimulating AL neurons with varying blend ratios while simultaneously recording the activity of PN pairs, it was shown that MGC-PNs produce peak correlations at the natural 1:2 blend ratio, and those correlations significantly deteriorate in response to stimulation with behaviorally sub-optimal blend proportions (Martin et al., 2013). Such stimulus-quality-affected correlations in the PN spikes were also reported for glomeruli other than those of the MGC, in experiments that manipulated the ratios of naturally-occurring hostplant blends (Riffell et al., 2009a).

The mechanisms determining spike correlations are unknown, but balanced inhibition may be involved. Upon pheromonal stimulation, both PNs and LNs are activated, with cPNs and tPNs excited by their cognate pheromone constituents and reciprocally inhibited through GABAergic LNs (Lei et al., 2002). LNs likely respond in a dose-dependent manner, allowing the inhibitory effect exerted onto PNs to be modulated by the relative proportion of the blend constituents. Moreover, the degree of spike coincidence between PNs is positively correlated with the strength of the inhibitory input onto those PNs (Lei et al., 2002). Similarly, in the AL of cockroaches, GABAergic LNs also mediate synchronization of PN outputs (Watanabe, 2012). Thus, balanced lateral inhibition is a plausible mechanism by which stimulation with a pheromone blend of optimal ratio can produce the highest degree of correlated spikes in PNs. These ideas are yet to be experimentally confirmed, but have already been explored to some extent in a modeling study (Zavada et al., 2011). Given the diversity of LNs in the AL (Wilson and Laurent, 2005; Seki and Kanzaki, 2008; Reisenman et al., 2011), lateral inhibition may involve particular LN types. Indeed, a recent study on the silkmoth *B. mori* revealed the existence of both spiking and non-spiking LNs, and showed that non-spiking LNs can inhibit PNs (Tabuchi et al., 2015). Some of these effects may be species-specific, as spiking LNs in the AL of the cockroach *Periplaneta americana* can inhibit PNs (Warren and Kloppenburg, 2014), while non-spiking LNs (at least those surveyed) do not (Husch et al., 2009).

If the observed spike correlations are meaningful, then the correlated code should be read by postsynaptic neurons. Indeed, although rare, some lateral horn protocerebral neurons, which are known to receive direct input from AL neurons and thought to mostly mediate innate behaviors (e.g., Homberg et al., 1989; Anton and Homberg, 1999; Jefferis et al., 2007; Galizia and Rössler, 2010; Roussel et al., 2014; Kohl et al., 2015), produce the strongest response to the two-component pheromone blend presented at the naturally occurring ratio (Lei et al., 2013). Such correlation hypothesis is also supported by a recent study in *Drosophila melanogaster*. The odor-evoked spikes of PNs innervating a particular glomerulus (DA1) are highly correlated and provide converging input to their target neurons in the lateral horn (Jeanne and Wilson, 2015). Although the ligand of DA1-PNs is a single pheromone compound (cis-vaccenyl acetate), these experiments demonstrate that synchrony between PNs (arborizing in the same glomerulus in this case) occur, and could be related to coincident detection in post-synaptic neurons (Jeanne and Wilson, 2015). The identity of other *Drosophila* volatile pheromone compounds, and their processing circuits, were recently reported, although it is not yet known which mixtures are behaviorally significant in this species (Dweck et al., 2015).

In summary, both behavioral and neurobiological data indicate that not just the identity of the sex pheromone constituents, but also the constituents' ratios, are of paramount importance in mediating natural behavior. The neural mechanisms underlying the coding of ratios, particularly at the higher brain level, are still not fully understood. Because responses to sex pheromone mixtures are often species-specific, those mixtures represent an effective way to control specific species, which is much preferable to the use of insecticides as these often affect non-target species.

### Use of other pheromones

In this section we focus on aggregation and alarm pheromones, since those are the only non-sex pheromone types that have been used to manipulate olfactory behavior. We will briefly review what is known for the major groups of harmful insects.

Aggregation pheromones promote conglomerates of individuals and are ubiquitous among arthropods, including many harmful species of beetles, moths, thrips, triatomines, locusts, mosquitoes, sand flies, and ticks (Wertheim et al., 2005; Sonenshine, 2006; Cook et al., 2007; Lorenzo Figueiras et al., 2009). Often, the decay, fermentation and pathogenesis associated with insect aggregations are the cause of important economic damage to crops and goods (Wertheim et al., 2005; van der Goes van Naters and Carlson, 2006). For instance, all throughout North America pine forests have been succumbing to massive bark beetle infestations that destroyed expanse forests and increase the risks of mudslides and forest fires (Chapman et al., 2012; Raffa et al., 2013). Beetle aggregation pheromones have been used for monitoring and mass-trapping, and also to recruit large number of insects on trap trees that are then destroyed (see Cook et al., 2007 for a review). A recent study used single-sensillum recordings to investigate the odor response profiles of ORCs in both sexes of the brown spruce longhorn beetle *Tetropium fuscum*. Interestingly, it was found that the responses to aggregation pheromones and plant volatiles are not completely segregated and can be synergized by the presence of volatiles indicative of host stress (MacKay et al., 2015).

While in general aggregation pheromones attract both sexes (Wertheim et al., 2005), in some species gravid females are attracted to a pheromone that induces aggregated oviposition. For instance, females of the sandfly *Lutzomia longipalpis*, which transmit leshmaniasis, use an oviposition aggregation pheromone which benefits the offspring of unrelated individuals by preventing fungal contamination of larval food (Wertheim et al., 2005). *Culex quinquefasciatus* gravid females, which are vectors of filariasis and West Nile Virus (among others), are attracted to a pheromone released from maturing eggs in conjunction with an indole compound derived from grass infusions (Mboera et al., 2000; Logan and Birkett, 2007), and these components evoke electrophysiological activity from antennal ORCs (Mordue et al., 1992; Blackwell et al., 1993). In other non-insect arthropods such as ticks, which transmit Lyme disease, fecal components promote arrestment and aggregation, and tarsi contact chemoreceptors respond to some of these components (e.g., guanine) with extremely high sensitivity (Grenacher et al., 2001; Sonenshine, 2006). Such information about the most effective bioactive components can have practical applications for tick control. For instance, aggregation pheromones can be used together with an acaricide that when applied to vegetation or livestock kills ticks upon contact (Sonenshine, 2006).

Alarm pheromones inform or alert a conspecific about impending danger; they are highly volatile, disperse quickly, and do not persist long (see Napper and Pickett, 2008 for a review). They are released by a variety of glands and include compounds belonging to different chemical classes (e.g., terpenes, hydrocarbons, nitrogen compounds). In blood-sucking insects, alarm pheromones could be used as repellents. Bed bugs release alarm pheromones in response to injury and ant attacks, causing conspecifics to disperse (Levinson et al., 1974a). This alarm pheromone is species-specific to a certain extent, and consists of two major components detected by antennal sensilla (Levinson et al., 1974b; Reinhardt and Siva-Jothy, 2007; Olson et al., 2009). When disturbed, adult triatomines release an alarm pheromone mainly composed of isobutyric acid that repels conspecifics (Guerenstein and Guerin, 2004; Manrique et al., 2006; May-Concha et al., 2013; Minoli et al., 2013a,b), which could be used as a triatomine monitoring tool (Minoli et al., 2013b). Isobutyric acid is detected by ORCs in grooved peg sensilla on the triatomine antenna (Guerenstein and Guerin, 2001), likely through the action of an IR (Guidobaldi et al., 2014).

Alarm signals are also conspicuously present in other hemipterans of economic importance such as stink bugs. Heteropteran alarm semiochemicals often have a six-carbon skeleton (e.g., trans-2-hexenal) and have little species specificity (Napper and Pickett, 2008). Insects of economic importance in other orders that produce an alarm pheromone include thrips and aphids. The alarm pheromone of thrips reduces oviposition and causes larvae to fall from plants, and thus could be used to pull insects away from crops (Pickett et al., 1997). When aphids are attacked, they release an alarm pheromone (trans-ß-farnesene; Bowers et al., 1972; Dewhirst et al., 2010; Vandermoten et al., 2012) that causes dispersion of other nearby aphids, including inter-specific responses across subfamilies (Napper and Pickett, 2008). This and other alarm aphid compounds have been used for controlling aphids in both greenhouse and field settings (Pickett et al., 1997; Dewhirst et al., 2010; Vandermoten et al., 2012).

Interestingly, sometimes a semiochemical can function as an alarm or an aggregation pheromone, depending on its concentration. This has been shown for trans-2-hexenal in cockroaches (Napper and Pickett, 2008), and for isobutyric acid in the blood-sucking triatomine bug *Rhodnius prolixus* (Guerenstein and Guerin, 2004; Manrique et al., 2006; Minoli et al., 2013a). Thus, not only the compound identity needs to be considered in tools for insect control, but also its concentration and behavioral context. While aggregation and alarm pheromones could be used to manipulate the olfactory behavior of harmful insects, we just started to understand how these signals are processed, particularly at the peripheral level. Control strategies can certainly benefit from a deeper understanding of the neural mechanisms controlling these olfactory-driven behaviors.

### Use of host odors

Many insects that feed or oviposit on a host such as a plant or a vertebrate are pests of crops or transmit human and/or animal diseases. It is well-established that host odors, including CO_2_, are a key cue for host detection and orientation (van der Goes van Naters and Carlson, 2006; Guerenstein and Hildebrand, 2008; McMeniman et al., 2014; van Breugel and Dickinson, 2014; Reisenman and Riffell, 2015). Much work has been done on the attraction of harmful insects toward natural and synthetic host odors and its neurobiological bases (Guidobaldi et al., 2014 and references therein), information that sometimes has been used to develop odor baits for traps (e.g., Krockel et al., 2006; Ryelandt et al., 2011; Mukabana et al., 2012; Guidobaldi and Guerenstein, 2013). Importantly, manipulation of host-seeking behavior offers many opportunities to disrupt harmful insects. Insects usually respond to specific mixtures of host odorants, even when they include ubiquitous (including non-host) odorants (Bruce and Pickett, 2011). Even when some constituents of those odor mixtures are essential to evoke a behavioral response (e.g., Geier et al., 1996; Guidobaldi and Guerenstein, 2013), in some cases certain components could have redundant roles and therefore, could be removed without decreasing attraction (e.g., Cha et al., 2008). Moreover, key components could be replaced without affecting attractiveness (Tasin et al., 2007). The neurophysiological bases of this phenomenon are not clear, but it is possible that in certain cases key odorants are detected by broadly tuned ORCs (that is, the same ORC could be involved in the detection of several behaviorally redundant key odorants). Thus, studies on the physiological responses of ORCs can have important implications for the design of attractive odor baits. Indeed, ORCs detecting different constituents of a natural odor mixture are sometimes co-localized in the same sensilla (Stensmyr et al., 2003). This, along with the finding that sometimes ORCs within a single sensillum interact (Nikonov and Leal, 2002; Ochieng et al., 2002, Su et al., 2012), makes possible the simultaneous detection and processing of mixture components already at the peripheral level.

As a general rule, odorant identities in the AL are encoded in spatial patterns of glomerular activation (Carlsson et al., 2002; Hansson et al., 2003; Wang et al., 2003; Lei et al., 2004), with some glomeruli narrowly tuned to certain odorants, including hostplant volatiles. For instance, PNs in a specific glomerulus of the *M. sexta* AL are extremely sensitive and narrowly tuned to the plant volatile cis-3-hexenyl acetate (Reisenman et al., 2005). Moreover, other PNs in a female-specific glomerulus can discriminate, with high sensitivity, the (+) and (−) enantiomers of linalool (Reisenman et al., 2004). PNs in sexually isomorphic glomeruli, in contrast, are equally responsive to both enantiomers of linalool (Reisenman et al., 2004). Interestingly, these neurophysiological findings served to predict behavioral responses that were readily tested. Thus, later studies found that the two enantiomers of linalool respectively mediate oviposition attraction and repellence (Reisenman et al., 2010, 2013), and that these two compounds are equally effective in mediating feeding (Reisenman et al., 2010).

Different features of host odor blends are encoded in glomerular activity patterns. For instance, the encoding of odor mixture identity involves synchronous firing of PNs throughout the activated glomeruli, which may serve to “bind” the components of the odor mixture (Riffell et al., 2009a,b). In addition, stimulation with an odor mixture can evoke a glomerular activation pattern which is different from that evoked by the summation of the activity patterns evoked by each component (see below). The importance of ratios in the detection of host odor mixtures has been shown in different insects (e.g., Najar-Rodriguez et al., 2010; Guidobaldi and Guerenstein, 2016). In oriental fruit moths, for instance, particular ratios within a synthetic plant odor mixture affected oviposition attraction negatively. Corresponding neurophysiological studies found that information about component ratios occurs non-uniformly across AL glomeruli, and that further processing takes place in higher-order brain centers (Najar-Rodriguez et al., 2010).

As mentioned above, insects usually respond to specific host odor mixtures (e.g., Geier et al., 1999a; Barrozo and Lazzari, 2004a; Krockel et al., 2006). For example, triatomines are sensitive to various human compounds (e.g., CO_2_, lactic acid, ammonia, carboxylic acids; Guerenstein and Lazzari, 2009), and a mixture of ammonia, lactic acid, and pentanoic acid evokes attraction, whereas there is low or no attraction to the single constituents (Guidobaldi and Guerenstein, 2013). Furthermore, in aphids, individual constituents of an otherwise attractive blend can have repellent effects (Webster et al., 2010). Some constituents of host odor mixtures can act synergistically to evoke attraction (e.g., Bosch et al., 2000; Barrozo and Lazzari, 2004a; Smallegange et al., 2005; Piñero et al., 2008; Guidobaldi and Guerenstein, 2013). In females of the oriental fruit moth *Cydia molesta*, minute amounts of benzonitrile added to an unattractive mixture resulted in a mixture that is as attractive as a natural blend. At the AL level, this bioactive mixture evoked strong activation and synergistic effects in an additional glomerulus not activated by the unattractive mixture (Piñero et al., 2008). Besides synergistic phenomena, additive effects in response to odor mixtures are also found at the central level (e.g., Lei and Vickers, 2008). Therefore, multi-component odor baits will likely be more attractive than single odorants, as they may form specific and reliable “odor objects” (e.g., Späthe et al., 2013, see Section Effects of Background Odor). Interestingly, it has been proposed that just a few (sometimes just three) key components of an odor blend are sufficient for reliable host recognition, even when the insects can detect a higher number of host odorants (Qiu et al., 2007; Riffell et al., 2009a; Guerenstein and Lazzari, 2010; Bruce and Pickett, 2011; Guidobaldi and Guerenstein, 2013).

CO_2_ is a food and/or oviposition host cue used by some herbivorous and hematophagous insects (Guerenstein and Hildebrand, 2008). Glomerulus-specific CO_2_ PNs in the AL of *M. sexta* can follow high frequency CO_2_ pulses, suggesting that these PNs report information about long-distance CO_2_ cues (Guerenstein et al., 2004a). This idea is also supported by the finding that nectar-rich flowers emit relatively high levels of CO_2_ (Guerenstein et al., 2004b). In fact, foraging moths use floral CO_2_ as a long-distance cue to find those flowers (Thom et al., 2004; Goyret et al., 2008). This and other examples (e.g., van Breugel et al., 2015) again show that neurobiological studies can predict behavior, and ultimately can inspire odor-based control strategies (van der Goes van Naters and Carlson, 2006). The fact that blood-sucking insects are proving difficult to control (Logan and Birkett, 2007), and that they transmit an ever increasing number of diseases to humans and animals, emphasizes that further studies are needed to develop effective tools for insect behavioral manipulation. It should be noted that any odor-based control strategy should consider that different types of natural odor stimuli (including background odors) often interact (e.g., Chaffiol et al., 2012, 2014, see also Section Effects of Background Odor). In addition, it should be considered that the physiological state of the insects (e.g., mating, feeding) as well as learning affects their responses to odors (e.g., Barrozo et al., 2010; Saveer et al., 2012; Reisenman, 2014; Matthews et al., 2016; Section Plasticity in the Responses to Semiochemicals).

### Combined use of pheromones and plant volatiles

When insects detect a mate, their olfactory system is confronted with not only sex pheromones, but also background odors such as plant volatiles. In principle, sex pheromones admixed with green leaf volatiles should be very attractive to phytophagous insects because such mixture may indicate the presence of a calling mate in a proper context. Therefore, at least in certain cases, it would be important to include hostplant volatiles in sex pheromone traps. For instance, in the case of the codling moth *Cydia pomonella*, addition of plant volatiles [e.g., (*E*)-β-farnesene] to the sex pheromone (codlemone) significantly increased the proportion of males flying to the pheromone in wind tunnel experiments (Schmera and Guerin, 2012; Trona et al., 2013). In addition, it has been shown that females of the Egyptian cotton leafworm *S. littoralis* exposed to cotton volatiles start calling earlier than females exposed to non-host volatiles, and that mating pairs exposed to these volatiles start mating earlier. Also, more males reach (or arrive nearby) the pheromone source when hostplants, rather than non-hosts, are present (Binyameen et al., 2013).

Integration of sex pheromone and plant volatile information may occur at the peripheral level. For example, in the noctuid moth *Agrotis ipsilon* pheromone ORCs can be directly excited by plant volatiles (Rouyar et al., 2015). Moreover, in pheromone-specific ORCs of *Helicoverpa zea*, stimulations with binary mixtures of sex pheromone and single hostplant odorants [either linalool or (*Z*)-3-hexenol] produce stronger responses than stimulation with the sex pheromone alone due to interactions between ORCs (Ochieng et al., 2002). Mixtures containing pheromone and plant odorants can also have a suppressive effect. For instance, in *S. littoralis*, herbivore-induced plant odorants can directly suppress the response of pheromone-specific ORCs (Hatano et al., 2015). Direct suppression has also been observed in *Heliothis virescences* males upon stimulation of pheromone-specific ORCs with a sex pheromone component and a number of plant volatiles (Pregitzer et al., 2012). Suppressive effects can also be due to interactions between ORCs (Andersson et al., 2010). Interestingly, in woodboring beetles (*T. fuscum*), some ORCs respond specifically to their aggregation pheromone, although other ORCs specifically respond to the aggregation pheromone combined with at least one plant compound (MacKay et al., 2015).

The olfactory sub-system dealing with the processing of sex pheromone signals has traditionally been considered as a specialized system different from the “main” olfactory sub-system dealing with the processing of host/food odors. This notion was strongly supported by the identification of pheromone-specific ORCs (Bray and Amrein, 2003; Mitsuno et al., 2008; Krieger et al., 2009; Grosse-Wilde et al., 2010; Montagné et al., 2012; Zhang et al., 2015) which in some insect species (particularly within Lepidoptera) project to a small but distinct number of male-specific glomeruli (the aforementioned MGC; Kanzaki and Shibuya, 1983; Christensen and Hildebrand, 1987; Hansson et al., 1992, 1995, 2003; Berg et al., 1998; Rospars and Hildebrand, 2000; Masante-Roca et al., 2002; Sadek et al., 2002; Lei et al., 2004). In spite of this anatomical and often functional separation, it is clear that the two olfactory sub-systems also interact at the AL level. Both suppressive and additive interactions between pheromone and plant odorants have been reported in the MGC of different Lepidoptera species. In some cases, suppressive effects were observed (Chaffiol et al., 2012; Deisig et al., 2012), while in others responses were enhanced (Namiki et al., 2008). The responses of neurons in sexually isomorphic glomeruli can also be affected by the presence of female pheromones in several species, but showed more interspecific variations (Namiki et al., 2008; Chaffiol et al., 2014). Moreover, in *C. pomonella*, both response enhancement and suppression in response to mixtures of pheromones and plant odors has been observed in sexually dimorphic and isomorphic glomeruli, respectively (Trona et al., 2013). Interactions between the two sub-systems are not necessarily reciprocal or determined by spatial proximity (Namiki et al., 2008; Reisenman et al., 2008; Trona et al., 2013). Furthermore, additive effects for single and pulsed stimulations with mixtures of pheromone and plant odors have been reported (Chaffiol et al., 2014). Because in most cases ORCs that respond to plant odorants do not respond to sex pheromones (and are located in different sensilla), the responses of AL neurons to sex pheromones in sexually isomorphic glomeruli likely result from AL network interactions (Reisenman et al., 2008; Deisig et al., 2012; Chaffiol et al., 2014). The processing of combined signals (i.e., pheromone and non-pheromonal) in higher brain centers is less understood, but it is likely that neurons in these centers further contribute to this interaction.

All these results, both at the peripheral (ORC) and AL level challenge the traditional idea that pheromone and hostplant odor reception and processing are segregated. Thus, these results indicate that olfactory neural circuits are perhaps far more functionally diverse than previously thought. At the same time, these findings highlight the idea that in order to develop efficient tools to manipulate mate-finding behavior it is important to consider the odor context of that signal (e.g., if appropriate for the species, pheromonal baits could also include a host odor).

Visual cues play important roles in modulating the olfactory behavior of insects (e.g., Green, 1986, 1993; Cardé and Gibson, 2010; Willis et al., 2011; Gaudry et al., 2012; McQuate, 2014; van Breugel et al., 2015), and thus, visual cues are often added to odor baits in traps (e.g., Green, 1994). As integration of visual and olfactory stimuli at the CNS has already been documented (e.g., Balkenius et al., 2009), further studies in higher brain centers could help improve the development of multimodal baits. Even when this integration of information is relevant for the manipulation of olfactory behavior, it exceeds the aim of this review, and will not be discussed here.

### Effects of background odor

Odor mixtures are thought to be represented in the insect brain as single “odor objects,” so that the unique mixture identity prevails over the information about its constituents (Lei and Vickers, 2008; Wilson and Sullivan, 2011; Stierle et al., 2013). When odor baits (usually odor mixtures) are used in the field for insect monitoring and control, they are necessarily presented against an odorous dynamic background (another odor mixture/s). Background odors can either be irrelevant, “mask” the target odor (making it unrecognizable), or can enhance the response to a target odor (Schroeder and Hilker, 2008). In principle, it is conceivable that the bait (target) plus the background odor are perceived as a single mixture, creating a new and emergent “odor object” that can interfere with the identification of the target odor. If that were the case, how do insects orient toward natural odor sources such as hosts, mates, and oviposition sites? In this section we review the importance of background odors in shaping the responses to a target odor bait.

Detecting and discriminating a target odor mixture requires binding its different components (e.g., Deisig et al., 2006; Riffell et al., 2009b), and this “odor object” should be salient even in the presence of background odors. How do nervous systems accomplish this task? In rats, prolonged odor stimulation leads to fast habituation of neurons in the olfactory cortex, so that new odors evoke clear, distinct, responses. As a result, when the two odors are present, the constant odor (background) is filtered while the target odor evokes a neural response, suggesting that animals can separate the target stimulus from its background (Kadohisa and Wilson, 2006; Linster et al., 2007). This idea is also supported by experiments in honeybees, in which odorants presented simultaneously (simulating components of a single odor source) were represented as a single object, while odorants presented with an inter-stimulus delay were represented separately (Szyszka et al., 2012; Stierle et al., 2013). Although interglomerular inhibitory interactions contribute to bind components into a single odor object (e.g., Deisig et al., 2006; Riffell et al., 2009b; Stierle et al., 2013), it has been shown that asynchronous mixtures activate more inhibitory interactions than synchronous mixtures (Stierle et al., 2013). How could this target-background object separation happen in natural odor plumes? Since insect ORCs can have short (<2 ms) response latencies, the thin filaments of target odors that intermingle with those of background odors could be resolved temporally, thus allowing target-background odor segregation (Szyszka et al., 2014).

Convincing and exciting experiments in moths showed that constant odor backgrounds that are chemically different from the target odor do not affect the representation of the target odor, whereas backgrounds that contain a constituent in common with the target odor do (Riffell et al., 2014), a phenomenon akin to the masking effect reported in mosquitoes and other insects (Logan et al., 2008; Schroeder and Hilker, 2008, see Section Odor Masking). Background odors with a constituent in common with the target evoke a change in the balance of excitation and inhibition in AL neurons with respect to the response to the target odor alone, thus altering the representation of the target odor (Riffell et al., 2014). Pre-exposure to this type of background odors produces an exacerbated change in the response to the target odor, resulting from neurons being adapted to the common constituent (Riffell et al., 2014). Stierle et al. (2013, see above) used a different insect species and different experimental conditions, although also tested dissimilar target- background odors presented simultaneously, and arrived to different conclusions (Stierle et al., 2013). These authors found that this mixture is represented as a single distinctive odor object, while Riffell et al. (2014) reported efficient target-background discrimination.

Still, there is an experimental situation that has not been tested yet: similar target- background odors (or target and background with a common blend constituent) presented asynchronously. Because in nature background odor plumes can have a different temporal structure than target odor plumes, insects could exploit these temporal differences to segregate a target odor from its background, even when these have common constituents (Stierle et al., 2013; Szyszka, 2014; Rusch et al., 2016). Experience may also help this segregation, as learning increases the distinction between different scents (Fernandez et al., 2009; Riffell et al., 2013). While in the work described synthetic blends were used, it would be most informative to use complete natural blends as targets since in principle, it should be easier to alter the neural representation of a synthetic mixture consisting of just a few constituents than that of a multi-component natural odor. Somewhat related to this idea, it has been suggested that redundant odor blends reduce uncertainty as they convey more robust information (Wilson et al., 2015).

As mentioned above (Section Combined Use of Pheromones and Plant Volatiles), plant odors could influence the response to pheromones both at the peripheral and the AL levels. Moreover, supression of attraction to the sex pheromone by hervivore-induced plant volatiles has been reported in *S. littoralis* (Hatano et al., 2015). However, *H. virescens* males can be effectively attracted to the conspecific female sex pheromone in a constant background of naturally-occurring hostplant odors, including hervivore-induced plant volatiles (Badeke et al., 2016). While these results parallel those reported by Riffell et al. (2014), the attraction of *H. virescens* to the female pheromone is impaired in a background of high and supra-natural plant odor concentrations (Badeke et al., 2016). These results not only further underlie the importance of using natural, realistic stimuli, but also that additional studies are necessary to fully understand the mechanisms underlying target/background discrimination, as the chemical identity of the odors used, as well as the species under study, could certainly influence the results.

A particular constituent of the volatile background, CO_2_, also affects the behavior of at least some insects (Guerenstein and Hildebrand, 2008). Information about this odor cue is processed as information about other odors, while the background level of CO_2_ is simultaneously encoded (Guerenstein et al., 2004a). In hematophagous insects this cue is used to detect and find vertebrate hosts (e.g., Geier et al., 1999b; Barrozo and Lazzari, 2004b), while in moths it is used to detect and find oviposition sites and nectar resources (Stange, 1997; Thom et al., 2004; Goyret et al., 2008). While those CO_2_ sources evoke clear responses from the CO_2_ ORCs at natural CO_2_ background levels, higher CO_2_ background levels interfere with those responses (Guerenstein and Hildebrand, 2008). In mosquitoes, an elevated CO_2_ background impedes take-off and source contact by masking the stimulus signal (Majeed et al., 2014). Moreover, the oviposition behavior of *Cactoblastis cactorum*, a moth particularly sensitive to CO_2_, is also affected by elevated CO_2_ backgrounds (Stange, 1997) because ORCs stop firing at such high CO_2_ levels (Stange et al., 1995). However, the behavior and ORC responses of *M. sexta* moths are not affected by moderate increases in CO_2_ background levels, but instead by high-amplitude CO_2_ oscillations (Abrell et al., 2005). In addition, certain background odorants can modulate the activity of CO_2_ ORCs (e.g., Guerenstein et al., 2004a) or even evoke a response *per se* in those receptors (Turner et al., 2011), thus interfering with CO_2_-mediated behaviors.

In conclusion, the odor background can affect responses to target odors (e.g., Büchel et al., 2014). Thus, for example, efficient odor baits developed in the laboratory could fail to attract insects under field conditions, where different background odors are present. Although more research is needed to understand its role in insect behavior, the odor background should be taken into account when planning an odor-based pest/vector management strategy. In addition, it would be important to investigate the feasibility of techniques to disrupt natural olfactory behavior using masking (see Section Odor Masking) and/or background odorants, as this could improve the methods currently used to disrupt behavior using natural odorants (see Section Disruption of Natural Olfactory Behavior).

## Olfactory repellence

According to Barton-Browne (1977) a repellent is “a chemical that acting in the vapor phase prevents an insect from reaching a target to which it would otherwise be attracted.” A repellent has also been defined as a product causing the insect “to leave the prospective host, with true behavioral repellency involving avoidance of the source of the repellent material, whether placed on the prospective host or near it” (Pickett et al., 2008). While these definitions are based on behavioral effects, the mechanisms of action of repellents are not considered. Repellents are used to stop a pest from finding a valued resource; topical repellents are usually applied onto the skin offering individual protection, while spatial repellents volatilize into the air, creating a vector-free space which provides protection for multiple individuals (Achee et al., 2012). Typically, volatile repellents are used to protect humans from insect (and other arthropod) bites, particularly from arthropods which are vectors of diseases (Foster and Harris, 1997). Repellents have also been used to protect crops: for example, the alarm pheromone of a number of aphids has been used against these pests (Foster and Harris, 1997; Pickett et al., 1997).

For centuries humans have used diverse parts of plants to repel biting insects (Moore and Lenglet, 2004). Among these so-called “botanical repellents,” various species of basil (*Ocimum* spp.) have been historically used to repel mosquitoes. In addition, oil extract from the leaves of neem (*Azadirachta indica*) has also been used as a personal mosquito and sandfly repellent (Yarnell and Abascal, 2004). Other botanical insect repellents include the oil from leaves of citronella (*Cymbopogon nardus*), palmarosa (*C. martinii martinii)*, lemongrass (*C. citratus*), and Eucaliptus (*Eucalyptus* spp.). The active components of these botanical repellents are often unknown although citral, a major ingredient in volatiles from lemongrass oil, and p-menthane-3,8-diol, from lemon eucalyptus, have repellent effects on a variety of mosquitoes (Yarnell and Abascal, 2004). Repellents can also be derived from other natural sources such as insects (as in the case of alarm pheromones or defense secretions), or may be purely artificial (Foster and Harris, 1997).

The world's most widely used synthetic topical insect repellent, with broad effectiveness against many insects, is *N,N*-diethyl-3-methylbenzamide, also known as N,N-diethyl-m-toluamide (DEET; White, 2007; Syed et al., 2011). Other synthetic repellents include Picaridin and IR3535 (or EBAAP, Ethyl Butyl-acetyl-aminopropionate). A full understanding of the mechanism of action of insect repellents and in particular, the identification of their molecular targets, can help design safer and more effective compounds. DEET appears to act both as a contact chemo-repellent that stimulates insect gustatory receptor cells that respond to aversive compounds (Lee et al., 2010), and as a volatile chemo-repellent acting on the olfactory system.

The mode of action of volatile repellents is still under debate and has been comprehensively reviewed recently (Leal, 2014); therefore, here we briefly summarize the most relevant investigations. In *D. melanogaster* and in the mosquitoes *Aedes aegypti* and *Anopheles gambiae* DEET appears to modulate the responses of ORCs to attractive odors (Davis and Sokolove, 1976; Ditzen et al., 2008). This effect depends both on ORCO (Ditzen et al., 2008) and on the molecular identity of the OR in the OR-ORCO complex (Pellegrino et al., 2011). However, for other repellents, it was proposed that DEET acts by just blocking ORCO (Tsitoura et al., 2015). On the other hand, Syed and Leal (2008) suggested that the mosquito *C. quinquefasciatus* can smell DEET directly and that that stimulation results in avoidance even in the absence of other odor cues. Similar results were reported in triatomines, suggesting a common mode of action for the repellent action of DEET (Zermoglio et al., 2015). Moreover, other additional findings further support the hypothesis that insects can smell DEET: (1) the existence of an ORC in *D. melanogaster* which is sensitive to DEET, picaridin and IR3535 (Syed et al., 2011) and, (2) electroantennogram (EAG) and single sensillum responses to DEET in *A. aegypti* (Stanczyk et al., 2010, 2013).

In an attempt to clarify some of these apparently contradictory results, Bohbot and Dickens (2010) characterized the effects of a number of repellents [DEET, 2-undecanone (2-U), IR3535 and Picaridin] on two OR-ORCO heteromers of *A. aegypti* individually expressed in *Xenopus* oocytes. Their results suggest that different mechanisms mediate the action of different repellents. That is, repellents could be smelled directly (acting as receptor agonists) or could inhibit the responses to odors (acting as receptor antagonists; Bohbot and Dickens, 2010).

It is now well established that insects can smell DEET (Leal, 2014). Studies in mosquitos suggest that ORCO and the OR pathway are necessary for the repellent effects of DEET as: (1) wild-type *A. aegypti* avoid DEET whereas ORCO mutants do not (DeGennaro et al., 2013) and, (2) in *C. quinquefasciatus*, different repellents activate a particular OR (*Cqui*OR136) in a dose-dependent manner, whereas knockdown of this OR resulted in loss of EAG and behavioral responses to DEET (Xu et al., 2014). These results suggest that an OR is involved in the direct detection of DEET (Xu et al., 2014). As the natural plant repellent methyl jasmonate elicits responses in ORCs expressing *Cqui*OR136, it has been proposed that this OR is tuned to natural repellents with long insect–plant evolutionary histories (Xu et al., 2014).

In summary, different hypotheses have been suggested to explain the mechanisms involved in the olfactory repellency of DEET in blood-sucking insects. They include: (1) DEET may silence ORs responsive to attractive odors, a hypothesis that has now little support; (2) DEET is detected by one or a few ORs; (3) DEET may act as a “confusant” by modulating the activity of many ORs. Although it is possible that more than one of these mechanisms act simultaneously, it is likely that they are species-specific. Because all these proposed mechanisms involve ORs, these are relevant candidate molecular targets for the development of new repellents (Leal, 2014). Thus, based on knowledge on the molecular receptors, more efficient and safer volatile mosquito repellents could be developed. The need to develop new repellents is emphasized by the finding that some populations of *A. aegypti* are insensitive to DEET (Stanczyk et al., 2010). Besides the repellent effects of DEET discussed above, application of DEET on human skin results in an altered host odor chemical profile due to a fixative effect of DEET, and that effect could also contribute to repellency (Syed and Leal, 2008; Section Odor Masking). Finally, certain constituents of non-host odors can act as arthropod repellents (e.g., interaction between cattle flies and heifers: Birkett et al., 2004; interaction between fruit flies and fruit: Linn et al., 2005; interaction between ticks and dogs: Borges et al., 2015), providing opportunities for the development of natural, safer repellents. It should be noted that the response to an attractive host odor blend can be manipulated by adding non-host odorants (e.g., Linn et al., 2005), and also by altering the proportions of one or more host odorants (Section Odor Masking), causing either repellency (avoidance), or masking (loss of attraction; Section Odor Masking).

## Disruption of natural olfactory behavior

### Mating disruption

The most common behavior that has been disrupted using semiochemicals is mating. This strategy has been used to eradicate insects that became resistant to pesticides, including pests of apples, peaches, cotton, and grapes (see Wyatt, 2003; Witzgall et al., 2010). The basic idea of mating disruption involves the broadcasting of a chemical signal similar to the sex pheromones of the target species. The first registration of a mating disruption product in the USA was for the pink bollworm (Brooks et al., 1979); currently there are more than 120 disruption products registered in the US. Mating disruption usually involves the release of large amounts of species-specific synthetic sex pheromones (e.g., Witzgall et al., 2010); these high concentrations often “overload” the insects' sensory system, interfering with the detection of the usually lower amounts of pheromone released by mating partners (Cardé, 1990, see below). Besides this traditional approach (see below), new techniques and approaches are being developed to improve efficacy. A new design, which is literally an auto-confusion disruption method, involves the application of electrostatically charged wax powder (dubbed Entostat) onto the cuticle of male moths. Because the powder can be loaded with large quantities of female sex pheromone, male moths function as mobile dispensers. Indeed, Entostat-exposed codling moth males remained as attractive as a 0.1-mg pheromone lure for up to 24 h in laboratory experiments (Huang et al., 2010). The behavior of male moths that are normally attracted to natural sources of pheromone was completely disrupted after treatment with Entostat powder. Moreover, the males' ability to orientate to the pheromone lure remained significantly impaired 6 days post-application, arguing that Entostat augments the effect of sensory (peripheral) adaptation and CNS habituation (Huang et al., 2010).

According to Miller and Gut (2015), mating disruption methods can be broadly divided into two categories, i.e., non-competitive and competitive. Non-competitive methods involve interference with the sensory capabilities of males or females, or hampering pheromone emission, and examples include mating/calling suppression, camouflage, sensory imbalance, and desensitization. Competitive methods do not involve changes on the insects' sensory capabilities or on pheromone emission and, therefore, insects can respond equally well to other insects and trap lures. Thus, several mechanisms can mediate pheromonal mating disruption, including loss of sensitivity in ORCs (sensory adaptation), loss of sensitivity at the CNS level (habituation), camouflaging of the female's odor trail, competition between dispensers and natural pheromone, and unbalanced components in the synthetic pheromone (Cardé, 1990). We next discuss sensory adaptation and habituation.

Stimulation with high concentrations of pheromones generally reduce the response sensitivity of pheromone ORCs (i.e., ORCs adapt to the stimulus), a phenomenon which can be quantified using EAG. For instance, in male oriental fruit moths, the EAG amplitude decreased as animals approached high emission-rate sources, and this reduction was correlated with upwind flight cessation (Baker and Haynes, 1989). In another moth species, long-lasting EAG adaptation after pheromone pre-exposure occurred over a range of pheromone dosages and lasted more than 10 min (Stelinski et al., 2005). There appear to be significant species-specific variations in the capability of the olfactory system to adapt to pheromones. For instance, *Grapholita molesta* moths have a three-fold greater level of sensory adaptation after pre-exposure than *Choristoneura rosaceana* (Trimble and Marshall, 2010), a finding which may explain why *G. molesta* is readily more controllable using mating disruption than *C. rosaceana*. The mechanisms underlying sensory adaptation were investigated in the moth *M. sexta*. After presentation of an adapting pheromone stimuli, and in response to the pheromone test stimulus, type I trichoid sensilla produced sensillar potentials of lower amplitude than those from non-adapted sensilla, while the pheromone ORC spike frequency of adapted sensilla was concomitantly lower (Dolzer et al., 2003). Furthermore, pheromone stimuli lasting several seconds strongly activated protein kinase C in pheromone ORCs, while minute-long stimuli elevated cGMP concentrations. These results indicate the existence of distinct intracellular signaling mechanisms mediating short-term and long-term adaptation (Dolzer et al., 2008).

In order to produce habituation in AL neurons and, therefore, disrupt behavior, unnaturally high stimulus concentrations and/or frequencies can be used. In AL PNs, pheromone stimulation typically produces a burst of action potentials followed by an after-hyperpolarization (AHP) inhibitory phase (Christensen and Hildebrand, 1988; Lei et al., 2009). The AHP is critical to enable PNs to resolve intermittent stimuli, which is a universal feature of natural odor plumes (Murlis et al., 1992; Lei et al., 2009). Within a certain range of stimulus frequencies, PNs respond with a burst of action potentials (followed by a short AHP) to each odor pulse, faithfully reporting the temporal structure of the stimulus train. However, when the pulsing rate exceeds the response range of PNs (>10 Hz), neurons can only respond with a single burst of action potentials followed by a prolonged AHP (Christensen and Hildebrand, 1988; Lei and Hansson, 1999; Heinbockel et al., 2004). In addition, the excitatory and inhibitory phases can be both habituated by high stimulus concentrations. Increasing stimulus concentrations decreases the delay to the onset of the excitatory phase and increases firing rate eventually reaching saturation (Heinbockel et al., 2004; Fujiwara et al., 2009), while also decreases the delay to the onset of the inhibitory phase and increases its duration. In the upper range of concentrations, PNs only produce a brief (high-rate) burst that is followed by a lengthy AHP, which is similar to the habituating pattern evoked by high frequency stimuli. Thus, under sustained stimulation and high concentrations, PNs show responses which are not likely linked to natural behaviors. Because PNs also receive input from LNs, these may also contribute to PN habituation, as observed in *D. melanogaster* (Seki et al., 2010). Because many LNs are GABAergic and can therefore inhibit PNs (Hoskins et al., 1986; Christensen et al., 1993; Wilson and Laurent, 2005; Seki and Kanzaki, 2008), LN habituation would produce sustained PN disinhibition, potentially interfering with triggering natural behavior. Although the roles of LNs are still being investigated, it is thought that they may render the response of some PNs concentration-independent (e.g., Asahina et al., 2009; Olsen et al., 2010). In summary, investigations on sensory adaptation and habituation can be helpful to find the most effective chemicals that can be used to disrupt mating.

### Odor masking

As mentioned above (Sections Use of Sex Pheromones and Use of Host Odors), not just the identity of the constituents of an odor mixture but also their proportions (ratios) are important for attraction. For instance, humans are differentially attractive to mosquitoes and this could be due to individual host odor mixture variability (Logan et al., 2008 and references therein). In some cases low attractiveness has been linked to low levels of some odors. For example, in *A. aegypti*, addition of lactic acid to the skin of formerly unattractive humans can increase their attractiveness (Steib et al., 2001). Low or no-attractiveness to a natural host odor blend could also result from higher-than-normal concentrations of a natural constituent of the attractive blend (e.g., Birkett et al., 2004; Logan et al., 2008, 2009), a phenomenon attributed to blend repellency or masking (see also Section Effects of Background Odor).

Comparisons of the odor profiles of individuals with different attractiveness revealed that a few compounds are present in higher relative amounts in less-attractive individuals, including 6-methyl-5-hepten-2-one (Logan et al., 2008, 2009). When low and naturally occurring doses of this odor were added to naturally attractive human odor, upwind flight and probing were reduced. Although a repellent-blend effect can occur (Logan et al., 2009), a small increase in the amount (ratio) of a particular compound within the natural host odor mixture could also produce masking of the target odor so that the host is no longer recognized as such (Logan et al., 2008; see also Bruce and Pickett, 2011 for examples in phytophagous insects).

Many semiochemicals can be used in conjunction with other chemical tools in “push-pull” strategies. These strategies divert insects away from a valuable resource (the “push” away from, for example, a host) into an attractant (the “pull” component; Pickett et al., 1997; Cook et al., 2007). Masking odors could be used in push-pull control strategies to prevent host location (“pushing” insects away from the hosts) while at the same time, attractive odors could be used as baits in traps to “pull” the insects away from hosts (Cook et al., 2007; Logan et al., 2008). Neuroethology approaches could readily speed up the discovery of effective masking odors for use in control strategies. For instance, one strategy could be to test the degree of odor-object transformation in the AL (i.e., the change in the spatio-temporal response pattern of an ensemble of AL neurons) that is evoked by altered ratios of different compounds within the natural host odor mixture.

Carbon dioxide is an important odor that mediates the behavior of many harmful insects (Guerenstein and Hildebrand, 2008). Therefore, manipulation of the odors that modulate the response of the CO_2_ receptors (Section Effects of Background Odor; Turner et al., 2011), including inhibitory odorants that can mask human scent (Tauxe et al., 2013), can profoundly impact CO_2_-mediated behaviors. Moreover, large CO_2_ fluctuations can “confuse” the insect's detection of natural CO_2_ sources (Abrell et al., 2005 and references therein), which may be used for interfering with the behavior of CO_2_-sensing insects.

### Odor antagonism

As in many lepidopterans, *Heliothine* females release a sex pheromone that attracts conspecific males. However, certain compounds of the somewhat similar sex pheromone of a sympatric *Heliothine* species make the former blend unattractive. Indeed, the addition of such interspecific compounds to a species' sex pheromone blend can eliminate attraction in conspecific males, thus acting as antagonists (Vickers and Baker, 1997). In the AL of both *H. virescens* and *H. zea* the two essential components of their species-specific pheromone blends are represented in two separate MGC glomeruli. Odorants that antagonize attraction, when added to the respective pheromonal blends, evoked excitatory activity in PNs restricted to a third MGC glomerulus in both species (Vickers et al., 1998). Therefore, attractive and antagonist odor blends are represented in distinct combinations of MGC glomeruli, thus providing a combinatorial code for sex pheromone discrimination in sympatric species.

While approaching a female, male moths also emit volatile chemicals through specialized male structures such as the hairpencils (Birch et al., 1990). It has been shown that *H. virescens* hairpencil volatiles have both aphrodisiac and repellent effects on conspecific females and males, respectively. Interestingly, the male ORCs that respond to a conspecific hairpencil compound also respond to an interspecific sex pheromone antagonist (Hillier et al., 2006). Antagonist compounds (including both interspecific sex pheromone and conspecific hairpencil volatiles) are certainly amongst the important chemicals that can be used to manipulate harmful-insect behavior.

## Plasticity in the responses to semiochemicals

Behavioral plasticity (including associative and non-associative learning) affects chemosensory-guided behaviors in all insects. For simplicity, we define learning as a permanent change in behavior resulting from experience (Papaj, 2009). Associative learning involves pairing of two stimuli in a way that the response to one of the stimulus is altered as a consequence of the pairing, which is typically evaluated in classical/Pavlovian or operant/instrumental paradigms. For instance, a well-studied case of classical learning involves the pairing of an appetitive stimulus (e.g., sugar) that elicits a reflexive response (e.g., extension of the proboscis) with an odor; when an association between the two stimuli is formed, the sole presentation of the odor stimulus elicits proboscis extension (Bitterman et al., 1983). Behavioral habituation, a form of non-associative learning, reduces responsiveness to stable and repetitive stimuli, which can be important for detecting predators, food, and/or mate odors in an irrelevant and/or even complex olfactory background (Kadohisa and Wilson, 2006; Linster et al., 2007; Riffell et al., 2014; see also Section Effects of Background Odor). Behavioral sensitization is also a form of non-associative learning in which repeated presentation of a stimulus can result in amplification of responses to that and/or a related stimulus (Papaj, 2009).

Learning has profound effects on the chemosensory behavior of insects, including harmful ones. This is true even in the case of innate signals of prime biological relevance, such as sex pheromones. In moths, the action of sex pheromones depends on factors such as the presence of host-odors, sexual maturity, and mating status (Barrozo et al., 2011; Chaffiol et al., 2012, 2014; Guerrieri et al., 2012). Furthermore, moths can be trained to associate food with a sex pheromone (Hartlieb et al., 1999; Hartlieb and Hansson, 1999). In other cases, recognition of pheromones necessarily involves learning. In social insects, kin and nest-mate pheromones are learned by young larvae inside the nest, and maggot flies need to experience their own host-marking pheromone before they can discriminate between an occupied and an unoccupied fruit in which to lay eggs (Roitberg and Prokopy, 1981). Furthermore, in phytophagous insects, this kind of olfactory learning can promote the transition to new hosts of agricultural importance (Prokopy and Papaj, 1988; Papaj and Prokopy, 1989).

The way in which plasticity affects many different behaviors in herbivorous insects has been recently reviewed (see Anderson and Anton, 2014). In herbivorous insects, both larval feeding and adult experience can affect olfactory-guided oviposition, mate choice, and feeding (Riffell et al., 2008; Thöming et al., 2013; Anderson and Anton, 2014; Carrasco et al., 2015). In moths, plant volatiles can enhance male orientation toward the conspecific female sex pheromone (Chaffiol et al., 2012, 2014; Guerrieri et al., 2012). The learning abilities of pest insects should be particularly considered in control strategies. For instance, a “trap crop” (which always represents a small proportion of the cropping area) might be completely inefficient if insects first find the profitable crop and prefer this over the trap crop (Cook et al., 2007). Thus, the selection of the most effective crop border plants is crucial, and this can be achieved by screening plant cultivars coupled with identification of behaviorally and electrophysiological bioactive volatiles (Schröder et al., 2015). Other cognitive processes, such as habituation, have important implications in the management of pest insects (Section Mating Disruption). In diamondback moths, exposure to non-hosts can increase oviposition preference toward these plants, perhaps leading to host range expansion (Zhang and Liu, 2006).

In the case of insects vectors of human and animal diseases, learning and previous experience can have important epidemiological implications for disease transmission (McCall and Kelly, 2002). For instance, mosquito host choice is influenced by prior foraging experience, which causes them to return to less-defensive hosts and to hosts where feeding was more successful (McCall and Kelly, 2002; Lyimo and Ferguson, 2009). Not only that, but variation in the physical and chemical properties of blood can influence fitness and cause host feeding preferences (see Lyimo and Ferguson, 2009 for details). Thus, it has been suggested that pathogen transmission can be reduced by altering host choice (Lyimo and Ferguson, 2009). Also, mosquitoes tend to return to the same villages, houses, host species, and oviposition sites (McCall and Kelly, 2002). Then, it is not surprising that research in this area has expanded in the last couple of years, and it is now clear that blood-sucking insects can indeed learn and form new memories (Kaur et al., 2003; Jhumur et al., 2006; Tomberlin et al., 2006; Bouyer et al., 2007; Sanford and Tomberlin, 2011; Vinauger et al., 2011a,b, 2013, 2014; Chilaka et al., 2012; Sanford et al., 2013). Classical and operant paradigms showed that blood-sucking insects can associate stimuli of different modality (thermal, odor, gustatory, visual) while searching for a host and selecting oviposition sites. In *A. aegypti*, the association between odorants and a thermal appetitive stimulus is odor-dependent (e.g., certain odors can be readily learned, others are untrainable, etc). Furthermore, associative learning can modify the aversive deterrent effect of DEET in both kissing bugs and mosquitoes (Stanczyk et al., 2013; Vinauger et al., 2014). Learning processes also affect the responses to odors which are crucial for survival (e.g., pheromones). In triatomine bugs, a brief exposure to the alarm pheromone produces sensitization and increases the tendency to respond, while long-term pre-exposure elicits behavioral habituation (Minoli et al., 2013a). In blood sucking insects, however, our knowledge on the neural mechanisms underlying the effects of experience on chemosensory responses is mostly restricted to the periphery, as we discuss below.

In both blood-sucking and herbivorous insects the activity of ORCs can be affected by experience (e.g., long-term odor exposure and sensory adaptation to deterrents; see Section Mating Disruption). Experience can also cause downregulation of olfactory responses according to the feeding/mating status, and the time of the day (e.g., Almaas et al., 1991; Fox et al., 2001; Takken et al., 2001; Glendinning et al., 2009; Saveer et al., 2012; Stanczyk et al., 2013; Anderson and Anton, 2014; Claudianos et al., 2014; Reisenman, 2014). In general, associative learning is not usually represented at this level, although recent work in honeybees revealed that olfactory memories downregulate the expression of specific ORs. Furthermore, these changes occurred after conditioning and concomitantly, the population activity of antennal ORCs (measured as changes in EAG responses) decreased after learning (Claudianos et al., 2014). In mosquitoes, a reduction in the EAG responses to DEET correlates well with a post-exposure reduction in behavioral sensitivity to this repellent (Stanczyk et al., 2013).

The mushroom bodies mediate behaviors affected by learning and experience (e.g., Fahrbach et al., 1998; Zars et al., 2000; Huetteroth et al., 2015). However, in fruit flies and honeybees, learning already produces changes in glomerular volume and in synaptic distribution and density (e.g., Winnington et al., 1996; Devaud et al., 2001; Brown et al., 2002; Sachse et al., 2007; Arenas et al., 2012), and can modify neural representations at the AL level (e.g., Faber et al., 1999; Chen et al., 2015), including glomerulus-specific neural plasticity (Rath et al., 2011). In moths, pre-exposure to the conspecific female sex pheromone increases the response of male PNs (Anderson et al., 2007), and associative learning with an appetitive cue causes recruitment of additional responsive neurons (Daly et al., 2001, 2004). Furthermore, learning of the scent of flowers which are profitable but are not innately preferred increases activity in AL neurons (Riffell, 2012; Riffell et al., 2013), and serotonin and octopamine are both involved in this process (Dacks et al., 2008, 2012). Experience might also have important effects facilitating segregation between a target odor and its odor background (see Section Effects of Background Odor), by modifying the balance of excitation and inhibition in AL neurons (Riffell et al., 2014; Szyszka, 2014; Chen et al., 2015). Noctuid moths switch their olfactory preference from food odors to egg-laying (e.g., cotton) odors following mating, and calcium imaging experiments demonstrated that this switch is due to changes in the representation of these odors across the AL glomerular array (Saveer et al., 2012). The mechanisms involving AL plasticity include modulation of the activity of ORCs by inhibitory interneurons (Ignell et al., 2009; Chou et al., 2010; Root et al., 2011), and neuromodulation by biogenic amines, neuropeptides and hormones (Nässel and Homberg, 2006; Dacks et al., 2008; Saveer et al., 2012).

In summary, experience and learning readily affect the odor oriented behavior of harmful insects through many neurophysiological mechanisms, which need to be considered in control strategies that include baits, repellents, use of trap crops, etc. Neurophysiological studies could help discover the most effective control methods; e.g., through high through-output screening of potential repellents that do not cause adaptation in ORCs.

## Conclusions

Odor sources are widely used to manipulate the behavior of harmful insects. In recent decades, the neurobiological bases underlying insect olfactory behavior started to be unraveled. The insect olfactory system is able to encode the quality, quantity, and temporal features of the odor stimuli. Information about odor mixtures is also encoded, including the ratio between their components and discrimination in complex backgrounds. Moreover, responses to odors are modulated by the animal's internal and external state, and by experience and learning. Natural odors are usually odor mixtures (against a “noisy” background), and are represented as particular odor objects in the AL. Those odor objects signify relevant odor sources such as a host or a conspecific that, at least in some cases, could be “mimicked” in a simplified way using synthetic compounds, e.g., a male moth can be lured into a trap using synthetic versions containing few sex pheromone constituents. This facilitates the development of relatively simple and long-lasting odor baits to manipulate insect behavior. The simplified and optimal imitation of a natural odor mixture is challenging because it requires using only key mixture constituents, and this sometimes includes minor components within the natural mixture. Insect behavior can also be manipulated using repellents or “confusants.” The studies mentioned in this work and others are helping us to understand how the olfactory system processes information about odors, making possible to design very efficient odor baits, repellents, or ways to confound the insects. Moreover, those studies also generate predictions about natural olfactory behavior that are useful to devise odor-based strategies for insect control. Clearly, the fields of neuroethology and insect control could certainly benefit from reciprocal interactions, which need to be fostered by all partners involved, including funding agencies. Encouraging new steps are being taken in this direction such as a recent initiative between different agencies on the beneficial and antagonistic interactions between plants (including agricultural plants) and their pathogens (including insects). We hope that the information provided in this review will help find gaps in the knowledge about the neural bases of olfactory behavior that are worth filling, encourage related studies, and promote the application of existing information in the development of better methods to manipulate insect behavior for control purposes.

## Author contributions

PG contributed the general idea, wrote several sections, corrected the whole manuscript, and prepared the final version. CR wrote several sections, made several general suggestions, corrected the whole manuscript, and prepared the final version. HL wrote several sections, made general suggestions, and corrected the whole manuscript.

### Conflict of interest statement

The authors declare that the research was conducted in the absence of any commercial or financial relationships that could be construed as a potential conflict of interest.
